# Influence of long-term intensive use of irrigated meadow-chernozem soil on the biological activity and productivity of the arable layer

**DOI:** 10.1038/s41598-022-18639-1

**Published:** 2022-08-29

**Authors:** Natalia Nikolaevna Shuliko, Olga Fedorovna Khamova, Artem Yur’yevich Timokhin, Vasiliy Sergeyevich Boiko, Elena Vasilevna Tukmacheva, Anna Krempa

**Affiliations:** Omsk Agrarian Scientific Center, Omsk, Russia

**Keywords:** Microbiology, Environmental sciences

## Abstract

The research was carried out on the territory of the Russian Federation in the forest-steppe region of the south Western Siberia (Omsk state), in the long-term (43 years) stationary experiment. Sprinkling was used for irrigation in the experiment. The number of different physiological groups of microorganisms, the cellulolytic activity of the soil, and nitrification capacity were determined under the sowing of an eight-field grain-grass crop rotation (perennial grasses (*Bunias orientalis L.* + *Bromopsis inermis L.* + *Galega orientalis Lam. 6–8 years old),* spring barley *Hordeum vulgare Leyss.*—variety Sasha). Immobilization processes predominated in the soil under the sowed crops, it contributes to the preservation of soil organic matter (mineralization coefficient SAA/MPA < 1). The highest transformation ratio of soil organic matter, i.e. increased conversion of plant residues into organic matter, was noted with applying nitrogen-phosphorus fertilizers (N_60_P_60_) under the barley. The combination of irrigation factors and the use of mineral fertilizers (N_30-60_P_60_) were contributed to the growth of the microorganisms’ population, the amplification of decomposition of cellulose, and improvement of nitrification capacity in the soil. The perennial irrigation of the meadow-chernozem soil and the application of intensive technology of cultivation of crops in crop rotation stimulated the growth of the microorganisms’ population and didn’t detriment the ecological state of the soil.

## Introduction

The application of mineral fertilizers is the most effective way to increase the yield and the nutrition of plants and soil microorganisms. At the same time, the microbiological regime of the soil most of all affects the efficiency of the use of fertilizers. Therefore, when the biological activity of soils is increased, the growth of yield increases too^1,2^.

Thus, excessive activation of the soil microflora can lead to negative ecological consequences: deterioration of biological and physicochemical properties of soil, mineralization of humus, increase of gaseous nitrogen losses during denitrification and nitrification, accumulation of nitrates in the soil, plants, and groundwater, destruction of the stratospheric ozone shield^2–6^. Long-term use of high doses of mineral fertilizers can cause the activation and growth of toxin-forming microbes^7^.

Mineral fertilizers, applied in moderate doses, activates the viability of microorganisms of various physiological groups. According to that, in soils, the number of aerobic and anaerobic nitrogen fixers, ammonifiers, cellulose-decomposing bacteria, actinomycetes, and fungi are increased^8–11^.

The climatic conditions, especially the rainfall in the vegetation period have a significant impact on the plant development and their yeild^12^. The lack of moisture reduces the availability of mineral nutrition elements, including phosphorus, and changes the potential mechanisms of its replenishment in the soil solution^13^.

Optimization of the water regime during irrigation causes changes in the agrochemical and biological properties of soils, which are enhanced by the application of fertilizers^14^.

The water regime of the soil has a significant impact on the diversity of soil microorganisms and the enzymatic activity of loamy soils. Water scarcity causes changes in the relative abundance of certain types of bacteria. Microbial communities have a high susceptibility to drought in sandy soils and an important role of exogenous organic matter in protecting microbial activity during periods of drought^15–17^.

During irrigation, the number of microorganisms, which mineralize nitrogen and carbon-containing compounds, increases in the soil microbial community (in particular, these are ammonifiers and oligonitrophils), and this indicate an increase in mineralization of the organic matter process. At the same time, the number of fungi decreases^18^.

Intensification techniques and inappropriate irrigation methods can lead to losses of soil organic matter (humus), contamination of groundwater and other degradation processes, as well as negatively affect crop yields^19–21^.

Based on this, the research aimed to study changes in biological properties and direction of soil-microbiological processes in irrigated meadow-chernozem soil in variants with long-term application of mineral fertilizers, the effect of agricultural practices on the yield of crops, and the assessment of the ecological state of the soil.

## Material and methods

The studies were carried out in 2015–2017 in an irrigated grain-grass crop rotation under crops: perennial grasses (*Bunias orientalis* L*.* + *Bromopsis inermis* L. + *Galega orientalis* Lam. *6–8 years old),* spring barley *Hordeum vulgare* Leyss.—variety Sasha; in variants of the experiment without fertilization and with a medium supply of mobile phosphorus (according to Chirikov, up to 100 mg/kg of soil)—control and N_60_P_60_ for perennial grasses and barley with a high content of P_2_O_5_—more than 150 mg/kg (fertilized).

Moisture control in heavy loamy soils is carried out in the range of the moisture content of capillary rupture to the lowest moisture capacity of the soil. The moisture content of the capillary rupture and the lowest moisture capacity of the soil in the 0–1.0 m layer is 210–220 mm and 290–300 mm, in the 0–0.6 m layer—115–120 mm and 150–160 mm, respectively. Irrigation rate was recommended according to the data of monitoring the moisture content in the soil in the layer 0.6 (1.0) m every 10 days and were carried out by the wide-coverage wheel sprinkler “Volzhanka” sprinkler machine. The irrigation rate was 300 m^3^/ha, determined by the weather conditions of the growing season. In 2015–2016 yrs. the irrigation rate on perennial grasses was 1200 m^3^/ha, on spring barley 300 m^3^/ha. In 2017 yr. the irrigation rate on perennial grasses was 1500 m^3^/ha, and on spring barley 600 m^3^/ha.

The soil was meadow-chernozem, medium-thick, medium-strength, heavy loamy with a humus content of 7% (according to Tyurin) in a layer of 0–0.045 m, the depth of groundwater is 2.5–3.0 m. Soil pH was neutral—7.0–7.2. In the layer of 2–4 m, the salt content varies by layers—from low to high salinity, sulfate-soda, or soda-sulfate chemism^22^.

Water source—Om’ river (Om’ river—a river in the Asian part of Russia, in Western Siberia, in the Novosibirsk and Omsk regions; a right tributary of the Irtysh). Mineralization of water in August was around 0.5 g/l, hydrocarbonate-chloride, calcium-magnesium chemism.

The depth of groundwater occurrence in the stationary zone in the initial period of research changed from 4.5 to 5.5 m, in 2015–2017 yrs. 25–3.0 m. Mineralization of groundwater from steal to low- and medium-mineralized, hydrocarbonate-sulfate or sulfate-hydrocarbonate, sodium-magnesium chemism, pH within 7.1–8.5. The sum of exchangeable cations is 34.5–36.3 mEq/100 g of soil, the compound is dominated by calcium—93.8%, magnesium—6.1%, the proportion of sodium doesn’t exceed 1%, it’s a baseline^22^.

The initial supply of mobile phosphorus varied from low (according to Franceson) to medium (according to Chirikov), and the supply of potassium was high. The nitrate-nitrogen content was determined in the soil by the disulfophenol method—it varied from low to medium.

Samples were taken in sterile parchment bags for microbiological studies three times during the growing season. The samples were taken using 4–5 injections with a drill to a depth of 0–0.2 m. Counting of the number of microorganisms was carried out on the solid nutrient medium: the total number of saprophytic bacteria that decompose organic nitrogen-containing compounds in the soil—on the meat-peptone agar (MPA); microorganisms that consume nitrogen in mineral form (NH_3_)—on the starch-ammonia agar (SAA). In case of differences of the yield level of variants, we considered the number of nitrifying microorganisms (a group of autotrophic (see autotrophs) microorganisms capable of obtaining energy for life through the oxidation of inorganic nitrogen compounds) on leached agar with the addition of double ammonium-magnesium salt of phosphoric acid, soil microscopic fungi on Czapek agar^2^.

Meat-peptone agar (MPA) is a nutrient-rich environment on which many heterotrophic microorganisms of various systematic and physiological groups develop: gram-negative, non-endospore-forming bacteria of the genera *Pseudomonas*, *Flavobacterium* and *Achromobacter*, gram-positive spore-forming rods of the genus *Bacillus rococina*, various cocci of the genera mycobacteria (genus *Mycobacterium*), some higher actinomycetes (genus *Actinomyces*–*Streptomyces*) and filamentous fungi (genus *Aspergillus, Penicillium, Trichoderma, Allernaria* etc.). Starch-ammonia agar (SAA) is a synthetic, solid, elective medium. On starch-ammonia agar, the number of microorganisms (bacteria and actinomycetes) assimilating mineral forms of nitrogen is revealed.

To determine the direction of soil microbiological processes, the ratios of SAA/MPA mineralization coefficient and MPA/SAA immobilization coefficient were calculated. The coefficient of transformation (L_M_) of organic matter was also calculated—the accumulation of humus substances in the soil L_M_ = (MPA + SAA) * MPA/SAA^23^.

The nitrification capacity of the soil and the potential accumulation of nitrate-nitrogen in favourable conditions for composting were identified according to Kravkow^24^. The rate of cellulose decomposition, as an indicator of the general biological activity, which depends on the soil and weather conditions, was determined in the field by the decomposition of cellophane film according to the application method of Tikhomirova^25^.

According to Kurakov, Guzev et al., when we assess the anthropogenic impact on the soil, one or two microbiological tests aren’t enough, because each test provides an answer to some question about the changes in the ecological situation^26^. The reliable assessment of the ecological conditions of irrigating soil with prolonged use of mineral fertilizers is possible with the use of the set of determinations of the abundance of the main systematic groups of microorganisms and their total activity by the intensity of cellulose decomposition and nitrification capacity.

For the assessment of biological activity of the soil that is known as the sum result of coupled biochemical processes was used the Azzy's method of relative values as presented by Karyagina^1^. The essence of the method is that for each biological indicator a relative assessment of its change in the variants of the experiment is given. In this case, the larger value is taken as 100. The relative values of the entire complex of biological characteristics are summed up for each option separately and based on the obtained data, we calculated a comparative score. The results of the research are processed by the methods of variance and correlation analysis^27^.

The growing season of 2015 had its features that influenced the growth, development of fodder, grain crops, and their harvesting. The increased temperature in April, May, and June accelerated the vegetation of perennial grasses, which pushed the first crop hay to an earlier date. The second half of the summer was characterized by a lack of heat, which slowed down the ripening of cereals. The increased temperatures in spring and at the beginning of the growing season were combined with good atmospheric moisture. The lack of precipitation in July was compensated by humidification in August, which in general balanced the ratio of heat and moisture during the growing season (hydrothermal coefficient 1.08). During the vegetation in 2016, 227 mm of precipitation fell at the norm of 197 mm (115% of the norm). In June-July, the amount of precipitation exceeded the norm by 74%. The temperature regime was increased. The hydrothermal coefficient in May–August was 1.09. In the growth of 2017, the air temperature was within normal limits with a lack of precipitation. The hydrothermal coefficient was 0.70, which indicates the dryness of the summer period. The largest amount of precipitation fell in July (66 mm—108%), during the growing season the amount of precipitation was only 144 mm (70% of the norm).

### Ethics approval

Materials comply with the code of ethics for scientific publications.

## Results

The application of mineral fertilizers on the irrigated soil stimulated the number of nitrifying bacteria and fungi to the greatest extent, by 27–52 and 77% respectively. The effect of mineral fertilizers on the soil microflora depended on the combined influence of factors of moisture and soil temperature, and the type of cultivated crop (Table 1).

**Table 1:** Biological activity of irrigated meadow-chernozem soil under perennial grasses at different levels of fertilization.

Indicator	2015 year		2016 year		2017 year		Average	
	Control	Fertilized	Control	Fertilized	Control	Fertilized	Control	Fertilized
Bacteria on MPA, million CFU/g	23.1 ± 1.2	25.8 ± 5.4	29.0 ± 6.8	30.0 ± 5.4	28.1 ± 4.5	45.0 ± 9.4	26.7 ± 1.4	33.8 ± 4.5
Microorganisms on SAA, million, CFU/g	29.3 ± 2.6	27.9 ± 5.4	26.2 ± 2.8	32.3 ± 7.8	18.6 ± 4.8	34.9 ± 7.5	24.7 ± 2.5	31.7 ± 1.6
Nitrifiers, thousand, CFU/g	1.90 ± 0.4	3.07 ± 0.8	1.57 ± 0.3	2.71 ± 0.4	1.43 ± 0.1	1.64 ± 0.4	1.63 ± 0.1	2.47 ± 0.3
Fungi, thousand, CFU/g	18.8 ± 5.0	25.7 ± 1.8	68.6 ± 2.2	77.7 ± 4.8	14.6 ± 5.1	15.8 ± 3.8	15.4 ± 1.2	27.2 ± 5.4
Decomposition rate of cellulose, %	39.0 ± 3.7	36.7 ± 3.2	59.3 ± 3.6	48.3 ± 1.5	39.4 ± 1.7	32.6 ± 7.3	45.9 ± 7.5	39.2 ± 4.8
Nitrification capacity, N-NO_3_, mg/kg	19.5 ± 1.4	26.4 ± 5.3	33.5 ± 6.2	25.6 ± 7.1	24.7 ± 2.5	21.0 ± 2.2	26.0 ± 5.2	24.3 ± 3.6
Total biological activity, %	100	106	100	110	100	126	100	114

Optimization of mineral nutrition during the cultivation of perennial grasses on an irrigated background in 2015 and 2016 to the greatest extent stimulated the growth of the nitrifying bacteria number—61.6 and 72.6% to the control, respectively. An increase in the number of nitrifiers indicates an intensive process of nitrification in the soil, and the formation of nitrates—the main source of nitrogen fertilization for plants, therefore, indicates an improvement in the nitrogen nutrition of the cultivated crop. Soil moistening in June-July 2016, when, in addition to the norm, 87.7 mm of precipitation fell, contributed to an increase in the number of soil fungi (68.6–77.7 thousand CFU/g). In 2017, under the influence of fertilizers, the number of microorganisms on MPA and SAA significantly increased by 60 and 87% compared to control, respectively. An increase in the number of microorganisms in the soil of fertilized variants associated with the enrichment of mineral nutrition elements, an increase in the amount of root litter during the growing season, and a large number of residues after harvesting crops on fertilized plots^26^.

On average, over three years of research, the application of fertilizers under perennial grasses on irrigation stimulated the number of nitrifying bacteria and fungi to the greatest extent, by 51.5 and 76.6% to the control. In the references, it was noted that an increase in the number of fungi can occur when the soil is acidified with fertilizers during their long-term use^26^. Since the main function of fungi is the decomposition of plant organic residues in the soil, it can be assumed that on fertilized backgrounds the process proceeds more intensively due to an increase in the amount of decomposed substrate.

The intensity of cellulose decomposition under perennial grasses did not differ significantly in the variants during the years of research. The difference between the variants was 2.3–11.0%. The cellulolytic activity was the highest (48.3–59.3%) in 2016, compared to 2015, 2017 yrs., which was associated with an increased temperature and heavy rainfall in June-July (163–187% of the norm).

The nitrification capacity of the soil in fertilized variants exceeded the control by 35.4% only in 2015; in subsequent years, the potential amount of nitrate-nitrogen was lower concerning the control on 17–24%. One of the possible reasons for it was a significant excess of the bluegrass component over the legume in the agrocenosis. In the control without nitrogen fertilizers, the share of the legume component was higher and the botanical composition of the grass mixture was more balanced. Under such conditions, the influence of perennial grasses on the biological properties of the fertilized soil in comparison with the control had a smaller positive effect. During the decomposition of bluegrass crops residues, the number and activity of microorganisms are lower and weaker than in leguminous plants^28^. Apparently, for the same reason, the intensity of cellulose decomposition in the fertilized variant in comparison with the control tended to decrease. However, the number of microorganisms in the soil during the application of fertilizers was higher in comparison with the control, which accordingly influenced the total biological activity of the soil. The total biological activity of the fertilized variants under perennial grasses was 6–26% higher than the control, depending on the conditions during the years of research.

The use of nitrogen-phosphorus fertilizers for spring barley under conditions of irrigation had a stimulating effect on the number of various groups of microorganisms (Table 2).

**Table 2:** Biological activity of irrigated meadow-chernozem soil under barley, depending on fertilization.

Indicator	2015 year		2016 year		2017 year		Average	
	Control	Fertilized	Control	Fertilized	Control	Fertilized	Control	Fertilized
Bacteria on MPA, million CFU/g	41.3 ± 6.1	39.0 ± 8.1	29.8 ± 6.3	33.3 ± 6.1	31.7 ± 1.6	56.4 ± 17.1	34.3 ± 2.6	43.0 ± 5.4
Microorganisms on SAA, million CFU/g	34.5 ± 3.6	33.5 ± 5.4	28.9 ± 3.4	31.5 ± 4.0	30.4 ± 6.7	60.9 ± 26.6	31.3 ± 1.3	42.0 ± 7.3
Nitrifiers, thousand CFU/g	2.04 ± 0.6	4.19 ± 0.3	2.79 ± 0.2	2.94 ± 0.3	1.96 ± 0.3	1.8 ± 0.2	1.49 ± 0.2	1.43 ± 0.5
Fungi, thousand CFU/g	18.2 ± 1.5	23.4 ± 7.3	24.1 ± 1.7	50.0 ± 12.1	18.8 ± 3.2	28.6 ± 2.6	20.4 ± 1.5	34.0 ± 6.3
Decomposition rate of cellulose,%	30.8 ± 0.8	63.6 ± 3.3	37.8 ± 4.2	64.7 ± 5.1	27.0 ± 1.2	40.7 ± 3.1	31.8 ± 2.5	56.0 ± 0.5
Nitrification capacity, N-NO_3_, mg/kg	19.0 ± 2.0	31.5 ± 2.4	24.5 ± 4.1	28.8 ± 2.8	21.8 ± 2.7	34.1 ± 3.6	21.8 ± 1.2	31.0 ± 1.2
Total biological activity, %	100	100	100	110	100	141	100	116

In 2015, the number of nitrifiers doubled in comparison with the control in a fertilized variant, in 2016–2017 yrs.—fungi by 107 and 52%, respectively. In 2017, the number of saprophytic bacteria on MPA increased by 78%, microorganisms on SAA increased by 100% (two times) concerning the control without fertilizers. On average, over the years of research, the application of mineral fertilizers significantly (by 66.6%) increased the intensity of cellulose decomposition in the soil, by 44.4%—nitrate accumulation in favourable conditions (soil nitrification capacity). At the same time, the total biological activity of the soil increased by 16%.

Thus, the long-term use of mineral fertilizers in stationary experiments in moderate doses (N_30-60_P_60_) for different crops had a stimulating effect on groups of soil microorganisms. In the average of the years of research, the number of nitrifying bacteria and fungi (by 52 and 76% to the control), and the number of saprophytic bacteria on the MPA and immobilizing NH_3_ on the SAA were extended (in 2017 year was 78 and 100% respectively).

The application of mineral fertilizers had a positive effect on the nitrification capacity of the soil—the amount of nitrate-nitrogen, which is formed due to the internal nitrogen resources of the soil, which made possible the potential for providing plants with available nitrogen (21–34 N-NO_3_ mg/kg). Under natural conditions, the supply of plants with nitrogen depends on the intensity of the nitrification process.

The intensity of cellulose decomposition in the irrigated soil upon application of mineral fertilizers fluctuated according to Zvyagintsev D.G. from medium to strong which indicates a high biological activity of the irrigated soil^2^. The calculation of the total biological activity under perennial grasses was higher by 6–26%, under spring barley by 10–41% relative to the control.

One of the important indicators of the biological processes activity in the soil is the ratio of microorganisms developing on starch ammonium agar (SAA) and meat peptone agar (MPA). An increase in this ratio indicates the predominance of the mineralization process in the soil and the intensive use of soil nitrogen, while its decrease indicates an increase in humification processes. The microbiological processes’ intensity of nitrogen-containing compounds transformation in the soil was estimated by the coefficients of mineralization (SAA/MPA) and immobilization (MPA/SAA)^23^.

The number of microorganisms on starch ammonium agar is associated with the consumption of mineral nitrogen. On average, over the years of research in the soil under crops, the processes of immobilization or fixation of nitrogen by microorganisms prevailed, the MPA/SAA ratio > 1. The soils under the pedoclimatic condition of Siberia have a high ability to fix the introduced mineral nitrogen, contributing to its preservation and accumulation in the zone of the root system. The continuous transformation of the immobilized nitrogen in the mineral products of decomposition allows us to provide plants with assimilable forms of nitrogen more evenly^29^.

The highest coefficient of organic matter transformation (L_M_ = (MPA + SAA) * MPA/SAA), which potentially reflects the intensity of the accumulation of humic substances in the soil, was under barley in variants with the application of mineral fertilizers (Table 3).

**Table 3:** Direction of soil-microbiological processes of irrigated meadow-chernozem soil depending on fertilization.

		SAA/MPA				MPA/SAA				Coefficient of transformation L_M_ = (MPA + SAA)* (MPA/SAA)			
		2015	2016	2017	2015–2017	2015	2016	2017	2015–2017	2015	2016	2017	2015–2017
Control	Perennial grass	0.66	1.27	0.90	0.92	1.51	0.79	1.11	1.08	70.5	41.4	61.3	55.6
	Barley	0.96	0.84	0.97	0.91	1.04	1.20	1.03	1.10	64.5	90.9	60.5	72.1
Fertilized	Perennial grass	0.78	1.08	1.08	0.94	1.29	0.92	0.93	1.06	103.1	49.4	57.9	69.2
	Barley	1.08	0.86	0.95	0.98	0.93	1.16	1.06	1.02	109.1	84.1	68.7	86.6
Least average difference LSD_05_ A		0.17				0.21				17.15			
LSD_05_ B		0.21				0.25				21.01			
LSD_05_ AB		0.29				0.36				29.71			

It should be noted that during the operation of the stationary experiment there was a reliable decrease in the humus content due to the removal of macronutrients due to the mineralization of processes, incl. nitrogen mineralized from humus since a moderate level of chemicalization in the previous period provided only partial compensation for nitrogen removal by the crop (soil content has been monitored since 1972 year). For the preservation of positive humus balance with using perennial grasses in crop rotations, especially bluegrass, on irrigated soils, it is necessary to apply organic fertilizers^30^.

**Figure 1:**
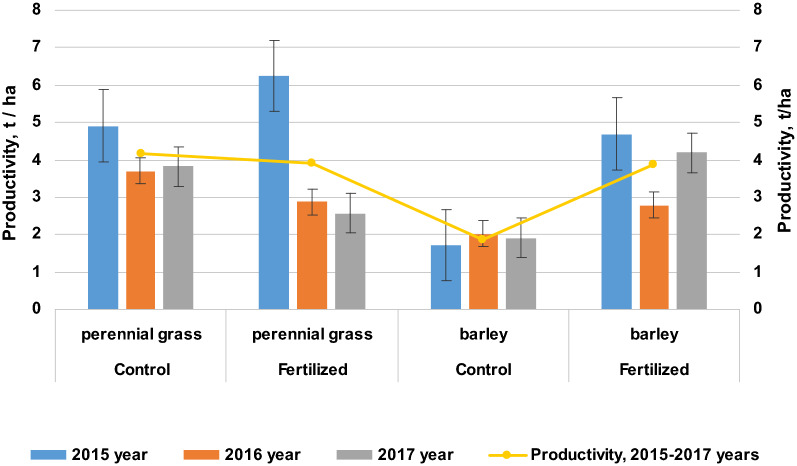
Productivity of crops depending on the use of mineral fertilizers, t/ha (2015–2017 years). On average for 2015–2017 optimization of soil moisture and conditions of mineral nutrition of plants contributed to an increase in barley yield by more than 100%, up to 3.88 t/ha. The collection of dry matter of perennial grasses during the study period in the fertilized version was at the control level due to a decrease in the share of the legume component with long-term use of nitrogen fertilizers.

The level of crop yields ultimately determines the effectiveness of the use of one or another agricultural technique. In 2015, the yield of crops in the crop rotation was higher in the variants with the use of fertilizers, an additional 2.97 t/ha of barley grain and 1.34 t/ha of dry matter of perennial grasses were obtained for two cuttings in comparison with the control (Fig. 1).

In 2016 and 2017 during the cultivation of barley, the same regularity was noted: on the fertilized variants the yield grain significantly increased by 0.76–2.28 t/ha. On average for 2015–2017 optimization of soil moisture and conditions of mineral nutrition of plants contributed to an increase in barley yield by more than 100%, up to 3.88 t/ha. In the cultivation of perennial grasses, systematic nitrogen fertilization displaced the *Galega orientalis* with *Bromegrass* and the yield of perennial grass dry matter was on average 0.25 t/ha less. The share of the cruciferous crop—*Bunias orientalis* was insignificant and did not affect the yield of the grass mixture.

Correlation analysis showed the presence of positive links of medium and strong degree between various indicators of the soil biological activity and the crops’ yield in a crop rotation with long-term irrigation (Table 4).

**Table 4:** Coefficients of correlation between various indicators of soil biological activity and crop yield, with n = 9, r crit. = 0.404.

Indicator of the soil biological activity	r	± Sr	t_f_	t_t_
**2016 year**				
Decomposition rate of cellulose, %	0.67	0.28	2.38	2.26
Nitrification capacity, N-NO_3_, mg/kg	0.91	0.16	5.8	2.26
**2017 year**				
Microorganisms on SAA, million CFU/g	0.46	0.33	1.37	2.26
Nitrification capacity, N-NO_3_, mg/kg	0.68	0.28	2.45	2.26

r—correlation coefficient, ± Sr—standard error of the correlation coefficient, t_f_, t_t_—criterion of the significance of the correlation coefficient (actual, theoretical).

In 2016, a strong link was obtained between the indices of the intensity of cellulose decomposition in the soil, nitrification capacity, and crop yield (r = 0.67 ± 0.28 and 0.91 ± 0.16, respectively). A similar connection of the crops yield with nitrification capacity was confirmed in 2017 (r = 0.68 ± 0.28). This was explained by the fact that in the process of nitrification, organic nitrogen-containing compounds under the action of microorganisms were mineralized to nitrate nitrogen, which was the main source of plant nutrition in Siberia^29,31^.

## Conclusion

Thus, long-term irrigation of meadow chernozem soil doesn’t decrease biological activities, and the use of intensive technology in crop rotation stimulated an increase in the number of soil microorganisms and their vital activity. Long-term irrigated meadow-chernozem soil with intensive cultivation of cereals (barley) had a high biological activity (up to 141% concerning the control), which positively affected the yield during the research years—2.78–4.69 t/ha and 1.72–2.02 t/ha on control.

The total biological activity in the arable layer of meadow-chernozem soil during intensive cultivation of perennial grasses was 106–126%. With prolonged irrigation in meadow chernozem soil under crops, the processes of nitrogen immobilization by microorganisms prevailed—MPA/SAA > 1.

Medium and strong correlations were established between indicators of the intensity of cellulose decomposition, the nitrification capacity of the soil, and the yield of cultivated crops (r = 0.67; 0.68–0.91, respectively) (Supplementary Information).

## Supplementary Information

Supplementary Information.
