# Author Correction: Influence of long-term intensive use of irrigated meadow-chernozem soil on the biological activity and productivity of the arable layer

**DOI:** 10.1038/s41598-022-23711-x

**Published:** 2022-11-17

**Authors:** Natalia Nikolaevna Shuliko, Olga Fedorovna Khamova, Artem Yur’yevich Timokhin, Vasiliy Sergeyevich Boiko, Elena Vasilevna Tukmacheva, Anna Krempa

**Affiliations:** Omsk Agrarian Scientific Center, Omsk, Russia

Correction to: *Scientific Reports* 10.1038/s41598-022-18639-1, published online 29 August 2022

The original version of this Article contained errors in the spelling of the authors Natalia Nikolaevna Shuliko, Olga Fedorovna Khamova, Artem Yur’yevich Timokhin, Vasiliy Sergeyevich Boiko, Elena Vasilevna Tukmacheva and Anna Krempa which were incorrectly given as Shuliko Natalia Nikolaevna, Khamova Olga Fedorovna, Timokhin Artem Yur’yevich, Boiko Vasiliy Sergeyevich, Tukmacheva Elena Vasilevna and Krempa Anna.

The original Article has been corrected.

