# Supplementary material for: Influence of long-term intensive use of irrigated meadow-chernozem soil on the biological activity and productivity of the arable layer
Source: Sci Rep. 2022 Aug 29;12:14672. doi: 10.1038/s41598-022-18639-1 (PMC9424210; doi:10.1038/s41598-022-18639-1)
Supplement: Supplementary file 1 — Supplementary Information. [file 41598_2022_18639_MOESM1_ESM.docx]

**Definitions, symbols, and abbreviations**

**Ammonifiers** - microbes that use proteins and other organic nitrogen compounds and break them down into amino acids. The amino group (NH2) is split off from the latter, from which ammonia (NH_3_) is formed. In ammonia, nitrogen is in the most reduced form, further nitrifying bacteria to nitrates and nitrites;

**biological activity of the soil** - the intensity of the ongoing biological processes;

**cellulolytic activity of the soil** (cellulose decomposition) - the ability of soil microorganisms to decompose cellulose (a polysaccharide with a high molecular weight, the main component of plant cell membranes);

**CFU/g** - colonies forming units per gram;

**fertilize**r - a substance designed to nourish plants and increase soil fertility;

**fungi** are a group of non-photosynthetic eukaryotic thallus organisms; they grow under aerobic conditions and receive energy by oxidizing organic matter;

**L_M_** - loss of humus due to mineralization;

**medium** - a substrate for the cultivation of microorganisms;

**mg/kg** - milligram per kilogram of soil;

**microorganisms** - the general name for the smallest organisms of plant and animal origin, visible only through a microscope. Microorganisms include bacteria, actinomycetes, yeasts, and moulds, as well as microscopic algae and protozoa;

**MPA** - meat - peptone agar;

**SAA** - starch - ammonia agar;

**saprophytic fungi and bacteria** are мicroorganisms that feed on the remains of plants and animals and convert organic matter into inorganic, thereby participating in the cycle of substances in nature;

**soil fertility** - a set of soil properties that ensure a harvest of crops;

**soil microflora** - a set of microorganisms present in the soil environment;

**stationary experiment** - a long-term experience with systematic fertilization, carried out on one site, in a crop rotation, or with a permanent crop;

**t/ha** - tons per hectare;

**yield** - average yield per unit of sown area.
